# Effects of Fermented Herbal Tea Residues on the Intestinal Microbiota Characteristics of Holstein Heifers Under Heat Stress

**DOI:** 10.3389/fmicb.2020.01014

**Published:** 2020-05-26

**Authors:** Yueqin Xie, Zujing Chen, Dongyang Wang, Guoping Chen, Xiaohong Sun, Qian He, Junyi Luo, Ting Chen, Qianyun Xi, Yongliang Zhang, Jiajie Sun

**Affiliations:** ^1^Guangdong Engineering & Research Center for Woody Fodder Plants, South China Agricultural University, Guangzhou, China; ^2^Guangdong Provincial Key Laboratory of Animal Nutrition Control, National Engineering Research Center for Breeding Swine Industry, College of Animal Science, South China Agricultural University, Guangzhou, China

**Keywords:** bacterial microbiome, heat stress, HTR, Holstein heifers, feed additive

## Abstract

Herbal tea residue (HTR) is a reusable resource with high nutritional value and bioactive substances content, which can be used as a feed additive. In the present study, HTRs were fermented by lactic acid bacteria, and then fed to a total of 90 Holstein heifers, termed as CN, LC, and HC groups. The supplementation improved physiological indices of respiratory frequency and rectal temperature, increased the concentrations of immunoglobulins and antioxidant capacity-related parameters, and reduced the concentrations of heat stress-related parameters and serum hormones. The heifers’ body height increased considerably, while their energy metabolism rates were stimulated in response to fermented HTRs. We also studied the fecal microbial community composition of 8 Holstein heifers in each group, and employed correlation analysis with tested parameters. We found that the bacteria were closely related to characteristics including the energy utilization rate, growth performance, serum biochemical indexes, and fecal SCFA levels of the heifers. Based on our findings, the 5% fermented HTRs replaced corn silage might be advantageous for the heifers’ characteristics under heat stress.

## Introduction

Herbal teas made from fresh or dried leaves, flowers, fruit, seeds, roots, and barks of various plant species have long been used as heat-clearing and detoxifying health care drinks in China (Li et al., 2017). In general, these herbal mixtures contain various biologically active compounds such as polysaccharides, flavones, organic acids, alkaloids, and volatile oils (Liu et al., 2011), which are involved in essential functions in humans and animals, including anti-oxidant, anti-inflammatory, anti-proliferative, anti-mutagenic, anti-bacterial, and anti-viral properties (Pardau et al., 2017). Thus, herbal teas are favored by people in the subtropical region of China because of these properties. Consumers believe that herbal teas have therapeutic effectiveness, are inexpensive, and have minimal or no toxic side effects compared with synthetic drugs (Abd et al., 2014). Thus, the demand for herbal teas has gradually increased and herbal teas are now produced on a large scale, which has led to increased production of herbal tea residues (HTRs). According to our survey, most producers treat HTRs by directly dumping or burning them, which not only poses a threat to the environment, but also wastes resources. In fact, HTRs can be recycled and reused. For example, HTRs can be used as a viable material for water purification, because they contain functional groups, such as carboxylate, aromatic carboxylate, phenolic hydroxyl, and oxyl, which have unusually high adsorption capabilities for toxic and harmful heavy metals (Yang and Cui, 2013). Many researchers have reported that tea residues could be used as adsorbent for Cesium [Cs (I)] (Gurung et al., 2013), Mercury (Hg) (Shen et al., 2017), and Chromium [Cr (VI)] (Ahsan et al., 2018). HTRs can also be used as composting materials to enhance soil fertility because of their high content of organic matter and nitrogen (Iqbal Khan et al., 2007). Moreover, some studies have shown that tea residues, because of their high content of polysaccharides and alkaloids, could be used as feed additives to improve the meat quality of goats (Zhong et al., 2009), change the gut environment of weaned piglets (Su et al., 2018), and improve the immune function in dairy cows under heat stress conditions (Shan et al., 2018). However, HTRs may be difficult to store, digest, and absorb because of their high content of moisture and fiber. This is a key issue when using them as feed additives. Therefore, the problem of how to store, digest, and absorb HTRs efficiently is attracting increased research attention. Microbial fermentation might improve the nutritive value, palatability, and digestibility of HTRs (Niba et al., 2009). A recent study confirmed that microbial fermentation also improved biological activities and exerted a greater immune effect on animals (Kim et al., 2017; Kumar et al., 2017). To date, however, no studies have focused on how to efficiently store and utilize HTRs. Therefore, in the present study, we aimed to develop a method to preserve HTRs and evaluated the possibility of using them as functional feed additives for Holstein heifers under heat stress.

## Materials and Methods

### Preparation of HTRs and Fermentation

HTR was obtained from Wong Lo Kat Limited (Guangzhou, China). It was cut into 2–3 cm pieces and mixed with oat hay (640:360 on a wet weight basis). The minor material, including 10% of corn flour, 2% of molasses, and 1% of lactic acid bacteria (*Lactobacillus plantarum GIM1.191*) (5 × 10^9^ colony forming units/g) were added and mixed thoroughly using a feed-stuff mixer. Finally, the HTR mixture was pressed into polyethylene bags (50 kg each) and fermented by anaerobic fermentation for 20 days.

### Nutritive Value Analysis

The samples of HTR and fermented HTR (days 0 and 20) were analyzed for dry matter (DM), crude protein (CP), ether extract (EE), and ash according to the AOAC International guidelines (Horwitz, 2010). The crude fiber (CF), neutral detergent fiber (NDF), and acid detergent fiber (ADF) contents were determined using the method reported by Van Soest et al. (1991). In detail, the contents of fermented HTR were determined from a water extract. Wet fermented HTR (20 g) was transferred to a glass bottle filled with 180 mL of deionized water, sealed, mixed, and stored at 4°C overnight (Fang et al., 2016). Then, the water extract was passed through filter paper and the filtrate pH was measured using a glass-electrode pH meter (Horiba D-21; Horiba, Tokyo, Japan). The lactic acid content of the filtrate was determined using the method of Barker and Summerson (1941).

### Animals, Experimental Design, and Treatments

The study was conducted in a scaled cow farm in Guangzhou, China. Ninety Holstein heifers (8 months of age and balanced for body weight) were used in a completely randomized design for a 7 days adaptation period and a 31 days experimental period. The heifers were randomly divided into three groups (30 heifers per group) in the same cowshed: CN group (no fermented HTRs or control, fed a basal diet); LC group (5% fermented HTRs replaced corn silage); HC group (10% fermented HTRs replaced corn silage). All heifers were fed a total mixed ration (TMR) twice daily. To meet their nutritional requirements, the TMR was based on corn silage as the main forage component and corn grain as the major concentrate component, according to Chinese feeding standards (China Standard NY/T34, 2004). The ingredients and nutrient composition of the HTRs, pre- and post- fermenting HTRs, and the three treatment diets are shown in Supplementary Table S1. All heifers were housed in an open sand-bedded cowshed, and water was available *ad libitum* throughout the experimental period. All animal procedures were approved by the Animal Care Committee at South China Agricultural University according to the university’s guidelines for animal research.

### Measurements and Sampling

The ambient temperature (AT) and relative humidity (RH) were recorded using a KTH-350-I temperature and humidity data-logger (Kimo Industry Co., Biarritz, France) at 08.00, 15.00, and 22.00 h. The temperature-humidity index (THI) was calculated as:

THI = (1.8 × AT + 32)–[(0.55–0.0055 × RH) × (1.8 × AT-26.8)] (Naderi et al., 2016). Respiration rates were determined by counting the number of flank movements in a 60-s period and were measured at 08.00, 15.00, and 22.00 h on Monday of each week (Srikandakumar and Johnson, 2004). Rectal temperature (RT) was measured immediately after respiratory rate (RR) observation using a 10 s digital thermometer (Digi-Vet SC 12, Kruuse, Langeskov, Denmark) that was inserted 8 cm into the rectum and determined at 08.00, 15.00, and 22.00 h (Kovács et al., 2018). The average daily feed intake (ADFI) was recorded on a daily basis. Body dimensions, including body length (BL), body oblique length (BOL), body height (BH), rump length (RL), and hip width (HW) were measured using a measuring stick and tape according to the method of Ozkaya and Bozkurt (2008).

On days 28–30, the diet offered to the heifers was sampled and used for nutrient analysis, chemical analysis, and gross energy (GE) measurement. The methods of nutrient determination, including CP, NDF, and ADFI, were consistent with the method described in Nutrition Value Analysis section. Chemical analysis of the calcium (Ca) and phosphorus (P) contents was performed using inductively coupled plasma spectroscopy (Chemists and Horwitz, 1990). The GE of the diet was determined using an automatic bomb calorimeter according to the method of Zou et al. (2016).

On the last experimental day, blood samples were collected from eight heifers in each group via the jugular vein before the morning feeding. Blood was placed on ice for more than 2 h and then centrifuged at 3,000 × *g* for 20 min at 4°C. The serum was stored at −80°C for further analysis of serum biochemistry parameters. Blood serum samples were analyzed for heat shock protein 70 (HSP 70), cortisol (Cor), lactate dehydrogenase (LDH), immunoglobulin A (IgA), immunoglobulin (IgG), alanine transaminase (ALT), creatine kinase (CK), total-antioxidant capacity (T-AOC), malondialdehyde (MDA), superoxide dismutase (SOD), and glutathione-peroxidase (GSH-PX) using the relevant commercial enzyme linked immunosorbent assay kits (Jiancheng Bioengineering Institute, Nanjing, China).

At the end of the experiment, we sampled feces from eight heifer rectums from each group and 400 g of feces per individual were collected at 08:00 h. One aliquot (100 g) was immediately mixed with 3 mL of 10% formaldehyde and stored at −20°C to determine fecal energy. The second aliquot (100 g) was used to determine nutrient apparent digestibility of DM, CP, NDF, and ADF using acid-insoluble ash (AIA) as a marker (Van Keulen and Young, 1977). The third aliquot (100 g) was used to determine fecal volatile fatty acids, including acetic acid (Aa), propionic acid (Pa), isobutyric acid (Ia), butyric acid (Ba), isovaleric acid (Iva), and valeric acid (Va) using high performance liquid chromatography analysis (Actlabs, Ancaster, ON, Canada). The fourth aliquot (100 g) was used to extract total genomic DNA. Total genomic DNA from feces was extracted using the cetyltrimethylammonium bromide/sodium dodecyl sulfate method. The DNA samples were tested for integrity using 1% agarose gel electrophoresis and their concentration was determined using a Qubit fluorometer (Invitrogen, Carlsbad, CA, United States). According to the concentration, DNA was diluted to 1 ng/μL using sterile water. The V3–V4 regions of the 16S ribosomal DNA (rDNA) genes were amplified by polymerase chain reaction based on the method of Sun et al. (2017). In details, the amplification was performed with the universal primers (forward primer, 341F: CCTAYGGGRBGCASCAG; reverse primer, 806R: GGACTACNNGGGTATCTAAT). Sequencing libraries were generated using an Thermofisher Ion Plus Fragment Library Kit (Thermo Scientific, Waltham, MA, United States) on an Thermofisher Ion S5^TM^ XL sequencer.

### 16S rRNA Gene Sequencing and Annotation Analysis

Single-end reads were assigned to samples based on their unique barcode in the adaptor sequence. Quality filtering of the raw reads was performed to obtain high-quality clean reads according to the Cutadapt quality controlled process (Martin, 2011). The reads were compared with the reference database (Quast et al., 2012) using the UCHIME algorithm (Edgar et al., 2011) to detect chimeric sequences (Haas et al., 2011), and clean reads were finally obtained using the Uparse software (Uparse v7.0.1001) (Edgar, 2013). Sequences with ≥97% similarity were assigned to the same operational taxonomic units (OTUs). For each representative OTU, the Silva Database was used to annotate taxonomic information based on the Mothur algorithm (Quast et al., 2012). To study the phylogenetic relationships between different OTUs, multiple sequence alignment was conducted using the MUSCLE software (Version 3.8.31) (Edgar, 2004). Alpha diversity was applied to analyze the complexity of species diversity within groups, including the Observed-species, Chao1, ACE, and Shannon indices. Beta diversity analysis was used to evaluate differences between groups using non-metric multi-dimensional scaling (NMDS). Two different complementary analyses including analysis of similarity (ANOSIM) and multiresponse permutation procedure (MRPP), were used to determine the significant differences of the fecal microbiota in response to fermented HTRs. All these indices were calculated using the quantitative insights into microbial ecology (QIIME) pipeline (Version 1.7.0) and displayed using the R software (Version 2.15.3). The raw sequences were deposited into Sequence Read Archive (SRA) database^1^ with the BioProject accession number PRJNA624971.

### Statistical Analysis

All data, including physiological parameters, serum biochemical indices, growth traits, energy metabolism rates, and the fecal concentrations of short-chain fatty acids (SCFAs) were analyzed using one-way analysis of variance (ANOVA) and Duncan’s test (SPSS 17.0, IBM Corp., Armonk, NY, United States). The R software was used to perform Metastat analysis to determine the differences in the relative abundance of fecal microbiomes (White et al., 2009). The correlation analyses of fecal microbiota with the tested traits were tested by function cor (x, y, use = “p”)^2^, and illustrated with function labeledHeatmap (Matrix, xLabels, yLabels) in R package WGCNA^2^.

## Results

### Chemical Compositions and Fermentation Quality of HTRs

The HTR had low DM, CP, EE, and ash contents of 205.88, 97.75, 35.17, and 66.85 g/kg, respectively. Additionally, it had high CF, NDF, and ADF contents of 288.28, 612.18, and 452.88 g/kg, respectively. After twenty days of anaerobic fermentation, the pH value dropped from 5.60 to 3.72; and the DM, NDF, and ADF contents decreased from 441.0 to 436.2 g/kg, 567.8 to 510.0 g/kg, and 332.0 to 301.2 g/kg, respectively. Simultaneously, the acetic acid, CP, and ash contents increased from 0 to 21.09 g/kg, 79.2 to 82.3 g/kg, and 50.0 to 55.0 g/kg, respectively (Supplementary Table S1). Additionally, compared with that of the CN group, the LC and HC groups were formulated to contain similar content of Ca (5.8, 5.6, and 5.6 g/kg, respectively) and P (4.0, 3.9, and 3.9 g/kg, respectively), a lower content of CP (130.1, 128.2, and 127.9 g/kg, respectively), but a higher content of DM (554.8, 560.2, and 563.8 g/kg, respectively), NDF (467.2, 473.1, and 479.8 g/kg, respectively), and ADF (220.9, 235.8, and 241.9 g/kg, respectively) (Supplementary Table S1).

### Physiological Index, Energy Utilization Rate, Nutrient Apparent Digestibility, and Growth Performance

The mean THI values in the morning (08:00), afternoon (15:00), and evening (22:00) in the barn during the study were 77.9 (range 75.4–80.7), 82.1 (range 79.6–83.9), and 77.6 (range 75.8–78.6), respectively (Supplementary Figure S1). The overall mean THI was 79.2. The results for the physiological index and ADFI are shown in Table 1. The RR and RT were affected by fermented HTR (*P* < 0.05). In detail, compared with that in the CN group, the RR in LC and HC groups decreased (*P* < 0.05) at 15.00 h, to 51.75, 47.02, and 48.29 breaths/min, respectively. The RT in LC group was similar at 8.00, 15.00, and 22.00 h (39.02, 39.10, and 39.08°C, respectively), which was significantly lower than that in the CN group (39.13, 39.27, and 39.19°C, respectively) (*P* < 0.05). Additionally, no differences in the RT were observed between the LC and HC groups (*P* > 0.05). The ADFI in the CN, LC, and HC groups were 14.24, 16.34, and 15.05 kg/day, respectively. The ADFI was the highest (*P* < 0.01) in the LC group and decreased significantly (*P* < 0.01) in the presence of an elevated dietary fermented HTR content.

**TABLE 1:** Food intake, respiratory rate, and rectal temperature of Holstein heifers fed HTR.

**Item**	**Time**	**Groups**		
		**CN**	**LC**	**HC**
Dry matter intake (kg/day)		14.24 ± 0.12^C^	16.34 ± 0.21^A^	15.05 ± 0.18^B^
Respiratory rate (breaths/min)	8:00	46.54 ± 2.81	45.75 ± 2.13	45.66 ± 2.11
	15:00	51.75 ± 1.79^a^	47.02 ± 2.26^c^	48.29 ± 2.73^b^
	22:00	46.44 ± 2.15	46.04 ± 1.68	46.17 ± 1.64
Rectal temperature (°C)	8:00	39.13 ± 0.31^a^	39.02 ± 0.18^b^	39.07 ± 0.25^ab^
	15:00	39.27 ± 0.21^a^	39.10 ± 0.25^b^	39.11 ± 0.27^b^
	22:00	39.19 ± 0.23^a^	39.08 ± 0.23^b^	39.14 ± 0.21^ab^

**The values were calculated as the means ± standard error of the mean (N = 30); a and b denote values that differ significantly at P < 0.05, while A and B denote values that differ significantly at P < 0.01. HTR, herbal tea residue; CN, control no HTR; LC, 5% fermented HTRs replaced corn silage; HC, 10% fermented HTRs replaced corn silage.**

The energy utilization rate is shown in Table 2. The DE/GE and ME/GE ratios in the CN, LC, and HC groups were 67.51 and 54.81%, 69.85 and 57.63%, and 69.22 and 57.77%, respectively. The values of DE/GE and ME/GE in the LC group were significantly higher than those in the CN group (*P* < 0.05). However, no differences in energy utilization rates were observed between the LC and HC groups. In addition, the apparent digestibility of DM, CP, NDF, and ADF was not different between the NC, LC, and HC groups (*P* > 0.05, Supplementary Table S1).

**TABLE 2:** Energy metabolism rate and quantitative analysis of short-chain fatty acids (SCFAs) in the feces of Holstein heifers among the three groups.

**Item**	**Groups**		
	**CN**	**LC**	**HC**
DE/GE (%)	67.506 ± 0.792^b^	69.851 ± 0.602^a^	69.219 ± 0.719^ab^
ME/GE (%)	54.812 ± 0.914^b^	57.634 ± 0.692^a^	57.774 ± 0.763^ab^
Acetic acid (mmol/L)	11.637 ± 0.153^C^	15.547 ± 0.154^A^	13.297 ± 0.329^B^
Propionic acid (mmol/L)	2.298 ± 0.034^c^	3.162 ± 0.029^a^	2.920 ± 0.123^b^
Isobutyric acid (mmol/L)	0.190 ± 0.013^b^	0.251 ± 0.007^a^	0.243 ± 0.005^a^
Butyric acid (mmol/L)	1.100 ± 0.044^c^	1.750 ± 0.037^a^	1.280 ± 0.073^b^
Isovaleric acid (mmol/L)	0.161 ± 0.005^b^	0.192 ± 0.005^a^	0.184 ± 0.006^a^
Valeric acid (mmol/L)	0.118 ± 0.004^b^	0.206 ± 0.007^a^	0.193 ± 0.003^a^

**DE, Digestive energy; ME, Metabolizable Energy; GE, Gross Energy; CN, control no HTR; LC, 5% fermented HTRs replaced corn silage; HC, 10% fermented HTRs replaced corn silage. The values were calculated as the means ± standard error of the mean (N = 30); a and b denote values that differ significantly at P < 0.05, while A and B denote values that differ significantly at P < 0.01.**

Results of growth traits measurements are shown in Table 3. The BH value was higher (*P* < 0.05) for the LC and HC group compared with that in the CN group. There was no significant difference between the LC and HC groups (*P* > 0.05). Other growth parameters including BL, BOL, RL, and HW were not affected by fermented HTR (*P* > 0.05).

**TABLE 3:** Analysis of serum biochemical indices in Holstein heifers between three groups.

**Item**	**Groups**		
	**CN**	**LC**	**HC**
HSP70 (pg/mL)	294.91 ± 5.20^A^	198.93 ± 7.24^C^	238.34 ± 3.64^B^
Cor (mg/mL)	116.30 ± 3.30^A^	76.52 ± 2.37^C^	104.64 ± 2.58^B^
LDH (U/L)	430.56 ± 8.51^A^	292.65 ± 8.09^C^	342.02 ± 8.28^B^
IgA (μg/mL)	215.11 ± 6.03^C^	317.14 ± 8.46^A^	280.15 ± 3.28^B^
IgG (μg/mL)	367.82 ± 7.32^C^	522.55 ± 6.04^A^	451.99 ± 12.89^B^
ALT (U/L)	4.54 ± 0.31^A^	2.92 ± 0.39^B^	3.06 ± 0.43^B^
CK (U/mL)	0.443 ± 0.062^A^	0.23 ± 0.021^B^	0.38 ± 0.041^A^
T-AOC (mmol/L)	0.167 ± 0.014	0.17 ± 0.018	0.17 ± 0.007
MDA (nmol/mL)	4.12 ± 0.47	3.76 ± 0.27	3.90 ± 0.19
SOD (U/mL)	78.30 ± 3.38^b^	94.80 ± 5.74^a^	82.43 ± 3.75^ab^
GSH-PX (U/mL)	100.23 ± 6.85^b^	121.61 ± 4.85^a^	120.05 ± 5.90^a^

**HSP70, Heat shock protein 70; COR, Cortisol; LDH, Lactate dehydrogenase; IgA, Immunoglobulin G; IgG, Immunoglobulin G; ALT, Alanine aminotransferase; CK, Creatine kinase; T-AOC, Total antioxidant capacity; MDA, Malondialdehyde; SOD, Superoxide Dismutase; GSH-PX, Glutathione peroxidase; CN, control no HTR; LC, 5% fermented HTRs replaced corn silage; HC, 10% fermented HTRs replaced corn silage. a and b denote values that differ significantly at P < 0.05, while A and B denote values that differ significantly at P < 0.01. The values were calculated as the means ± standard error of the mean (N = 8); a and b denote values that differ significantly at P < 0.05, while A and B denote values that differ significantly at P < 0.01.**

### Serum Biochemical Indexes

Serum concentrations of heat stress-related parameters (HSP70, Cor, and LDH), immunoglobulins (IgA and IgG), serum hormones (ALT and CK), and antioxidant capacity-related parameters (SOD, GSH-PX, T-AOC, and MDA) are listed in Table 4. For the heat stress-related parameters, the concentrations of HSP70 in the CN, LC, and HC groups were 294.91, 198.93, and 238.34 pg/ml, respectively, indicating a significant decrease (*P* < 0.01) when Holstein heifers were offered 5 or 10% fermented HTRs. The concentrations of Cor and LDH in the CN, LC, and HC groups were 116.30 mg/mL and 430.56 U/L, 76.52 mg/mL and 292.65 U/L, and 104.64 mg/mL and 342.02 U/L, respectively. The concentrations in the LC and HC groups were significantly lower than those in the CN group (*P* < 0.01). The concentrations of IgA and IgG were greater (*P* < 0.01) in the LC and HC groups than in the CN group. The concentrations of IgA and IgG in CN, LC, and HC groups were 215.11 and 367.82 μg/mL, 317.14 and 522.55 μg/mL, and 280.15 and 451.99 μg/mL, respectively. For the serum hormones, the concentrations of ALT in the CN, LC, and HC groups were 14.54, 2.92, and 3.06 U/L, respectively and the concentrations CK in were 0.443, 0.23, and 0.38 U/mL, respectively. The ALT level was significantly lower (*P* < 0.01) in the LC and HC groups compared with that in the CN group. Whereas the CK level in the LC group was lower (*P* < 0.01) than that in the CN and HC groups. For the antioxidant capacity-related parameters, the concentrations of SOD and GSH-PX in the CN, LC, and HC groups were 78.30 and 100.23 U/mL, 94.80 and 121.61 U/mL, and 82.43 and 120.05 U/mL, respectively. There levels in the LC group were significantly higher than those in CN group (*P* < 0.05). However, the concentrations of T-AOC and MDA were not affected (*P* > 0.05) by the fermented HTRs.

**TABLE 4:** Growth traits of Holstein heifers between three groups.

**Item**	**Groups**		
	**CN**	**LC**	**HC**
Body length (cm)	109.25 ± 0.55	113.13 ± 1.48	109.38 ± 2.17
Body oblique length (cm)	120.25 ± 1.16	126.25 ± 1.75	123.88 ± 3.02
Body height (cm)	113.13 ± 0.76^b^	117.00 ± 1.60^a^	117.25 ± 0.95^a^
Rump length (cm)	36.38 ± 0.86	38.75 ± 0.83	38.13 ± 1.61
Hip width (cm)	39.00 ± 0.26	41.38 ± 1.20	40.00 ± 1.95

**The values were calculated as the means ± standard error of the mean (N = 30), while a and b denote values that differ significantly at P < 0.05. CN, control no HTR; LC, 5% fermented HTRs replaced corn silage; HC, 10% fermented HTRs replaced corn silage.**

### Fecal SCFA Concentrations

The fecal SCFA concentrations are listed in Table 2. The concentrations of Aa in the CN, LC, and HC groups were 11.64, 15.55, and 13.30 mmol/L, respectively, with the LC and HC groups showing significantly higher levels than the CN group (*P* < 0.01). The concentrations of other fecal SCFAs, including Pa, Ia, Ba, Iva, and Va in the LC or HC groups were also significantly higher than those in the CN group (*P* < 0.05). However, no differences were found between LC and HC groups except for the concentrations of Pa and Ba.

### 16S rRNA Gene Sequencing and Annotation Analysis

**FIGURE 1:**
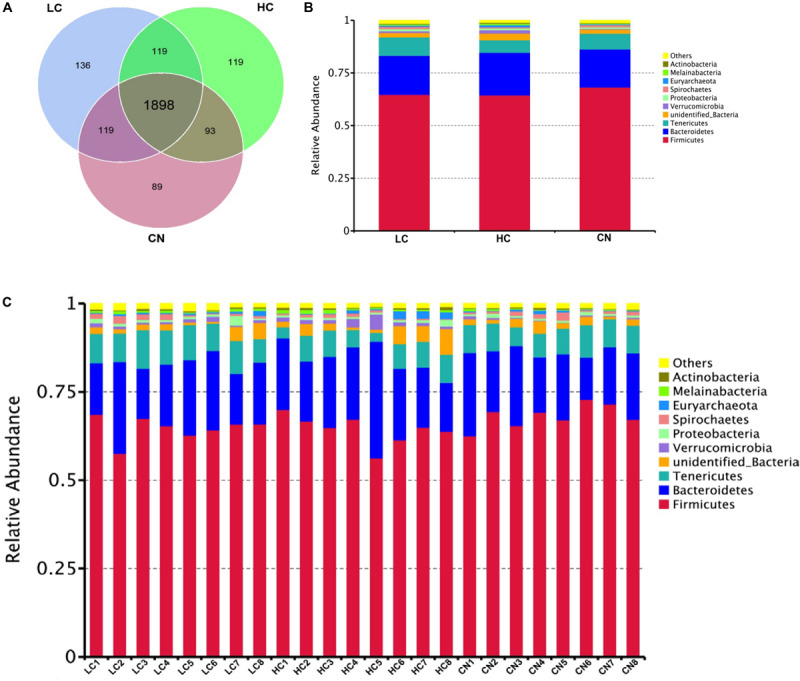
Common and specific OUT distribution of the fecal microbiota among three groups **(A)**; community bar-plots analysis shows relative abundance of fecal microbiota at the phylum level in each group **(B)** and in each sample **(C)**. CN, no herbal tea residues; LC, 5% fermented HTRs replaced corn silage; HC, 10% fermented HTRs replaced corn silage.

After DNA extraction, the hypervariable V3–V4 regions of the 16S rDNA were enriched, and a random rarefaction of sample reads was carried out to avoid errors caused by sequencing depth differences. The subsequent high-throughput analysis generated a total of 2,002,175 raw reads. On average, each sample produced approximately 83,423 joined tags (min = 70,458, max = 88,696). Over 94.33% ± 2.55% of the total joined tags from each sample passed quality control and were processed for further analysis (Supplementary Table S2A). Venn diagrams analysis of the high-quality tags yielded 2,573 unique OTU candidates at 97% sequence similarity, and 1,898 candidates that were shared across all samples were defined as core OTUs. The core OTUs comprised approximately 73.77% of the total candidates, while only 89, 136, and 119 OTUs were identified uniquely in the CN, LC, and HC groups, respectively (Figure 1A and Supplementary Table S2B). We annotated all these OTU tags to the Greengenes database, and found that 98.98 ± 0.25% of the sequences could be aligned at the phyla taxonomic level, while 98.12 ± 0.37%, 90.36 ± 2.11%, 82.95 ± 1.97%, 28.29 ± 4.02%, and 4.73 ± 1.64% of the annotated OTUs were assigned at the class, order, family, genus, and species levels, respectively (Supplementary Table S2C). Additionally, the microbial diversity in the feces of dairy cows was assessed using the QIIME pipeline based on the OTU annotation, which identified the top 10 phyla (Figures 1B,C). In detail, the most abundant phylum in the feces of Holstein heifers was *Firmicute*s, which accounted for approximately 65.79 ± 3.90% of all sequences, followed by *Bacteroidetes* (18.93 ± 4.57%), and *Tenericutes* (7.42 ± 1.94%) (Supplementary Tables S3A,B). At the class level, a total of 38 classes were detected, five classes had a relative abundance greater than 1.0%, including *Clostridia*, *Bacteroidia*, *Mollicutes, Erysipelotrichia*, and *unidentified_Bacteria*. The most abundant class in the feces of Holstein heifers was *Clostridia* (60.92 ± 3.84%) (Supplementary Tables S3C,D). At the order level, we detected four orders with a relative abundance greater than 1.0% (Supplementary Tables S3E,F). Specifically, the most abundant order in the feces of Holstein heifers was *Clostridiales*, which accounted for approximately 60.87 ± 3.83% of all sequences, followed by *Bacteroidales* (18.77 ± 4.39%), *Erysipelotrichales* (4.23 ± 1.18%), and *unidentified_Bacteria* (2.37 ± 0.65%). At family level, among the 137 families detected, eleven families had a relative abundance greater than 1.0%, including *Ruminococcaceae*, *Rikenellaceae*, *Christensenellaceae*, *unidentified_Clostridiales*, *Bacteroidaceae*, *Lachnospiraceae, unidentified_Bacteria*, *Peptostreptococcaceae*, *Erysipelotrichaceae*, *Muribaculaceae*, and *Prevotellaceae*, and the most abundant family in the feces of Holstein heifers was *Ruminococcaceae* (35.88 ± 3.73%) (Supplementary Tables S3G,H). Among the 257 genera detected, eight genera had a relative abundance greater than 1.0%, such as *Bacteroides*, Clostridiales, *Ruminococcaceae*, *Candidatus_Saccharimonas*, *Turicibacter*, *Alistipes*, *Romboutsia*, and *Paeniclostridium* (Supplementary Tables S3I,J). At the species level, among the 168 species detected, nine species had a relative abundance greater than 0.10%, including *Clostridium disporicum*, *Clostridium papyrosolvens*, *Methanobrevibacter ruminantium*, *Spirochaetes bacterium GWE2 31 10*, *Treponema porcinum*, *Ruminococcus bromii*, *rumen bacterium NK4A214*, *Pseudomona fragi*, and *bacterium LD2013*. The most abundant species in the feces of Holstein heifers was *Clostridium disporicum* (1.61 ± 0.22%) (Supplementary Tables S3K,L).

### Microbial Diversity in the Feces of Holstein Heifers

**FIGURE 2:**
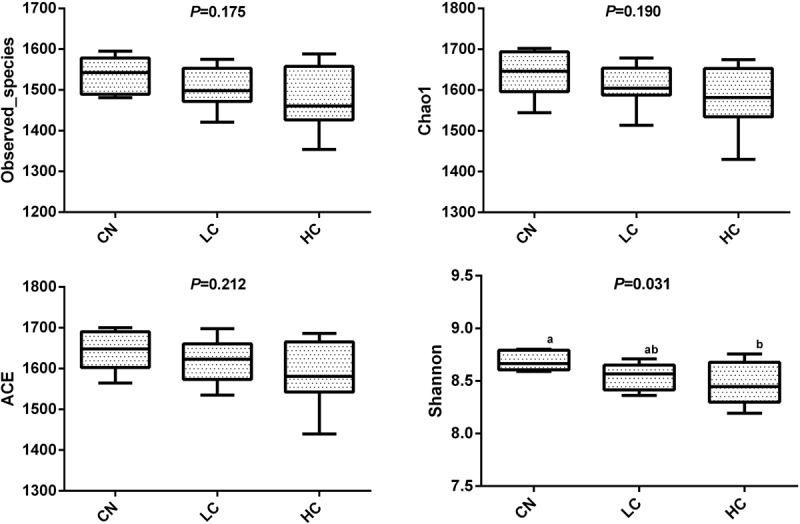
Microbial diversity indices in the fecal microbiome. Four measures of α-diversity include observed species, Chao 1 index, ACE and Shannon.

**FIGURE 3:**
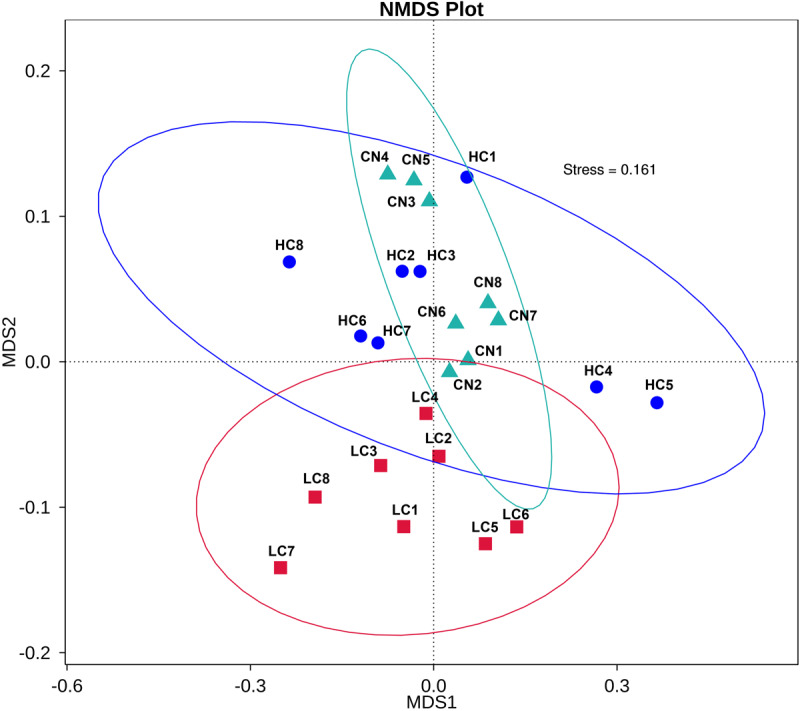
First two dimensions from the (non-metric) multi-dimensional scaling (NMDS) of the Bray-Curtis dissimilarity matrix. Each point in the figure represents a sample. The distance between points indicates the degree of difference, and the samples in the same group are represented by the same color. When stress is less than 0.2, it indicates that NMDS can accurately reflect the degree of difference between samples.

We compared the alpha-diversity (within-sample diversity or estimate of species richness and evenness) of each sample with differing sequence counts or sampling efforts. Rarefaction curve analysis indicated that the number of sequences and the sequencing depth was sufficient for this study (Supplementary Figure S2). We further used Observed species, Chao1, ACE, and Shannon indices among the different groups to evaluate the fecal diversity of Holstein heifers affected by fermented HTRs treatments (Figure 2). Although there was a trend toward decreased alpha diversity in the LC and HC groups compared with those of the control, these differences did not significantly affect the species-level microbial diversity as assessed using Observed species (*P* = 0.175), Chao 1 (*P* = 0.190), and ACE (*P* = 0.212). However, the Shannon diversity index showed a significant decrease in the diversity of the LC and HC groups compared with that of the CN group (*P* = 0.031). For the Beta diversity, NMDS showed distinct diversity differences between the groups (Stress = 0.161) (Figure 3). In addition, pairwise ANOSIM analyses suggested that there were extremely significant differences between the CN and LC groups (global *R* = 0.4905, *P* = 0.001), between the CN and HC groups (global *R* = 0.3245, *P* = 0.001), and between the LC and HC groups (global *R* = 0.2366, *P* = 0.008) (Table 5). We also performed an MRPP analysis in within- and between-group populations. The differences for between-group homogeneity were higher than those for within-group with an *A*-value > 0, and the change degree reached a significant level between the CN and LC groups (*P* = 0.001), between the CN and HC groups (*P* = 0.003), and between the LC and HC groups (*P* = 0.003) (Table 5).

**TABLE 5:** Significant differences in community structure in the fecal microbiota of Holstein heifers among the three groups.

**Group**	**Anosim**		**MRPP**			
	***R*-value**	***P*-value**	***A*-value**	**Observed delta**	**Expected delta**	***P*-value**
CN-LC	0.4905	0.001	0.09894	0.2821	0.3131	0.001
CN-HC	0.3245	0.001	0.0739	0.306	0.3304	0.003
LC-HC	0.2366	0.008	0.0746	0.3252	0.3515	0.003

**Anosim, analysis of similarity; MRPP, multiresponse permutation procedure. The R-value ranged from −1 to 1, and an R-value > 0 showed a significant difference in the between-group population compared with those in the within-group population, as well as the A-value (analyzed by MRPP, multiresponse permutation procedure). The P-values indicated the significant differences at the levels of P < 0.05 or P < 0.01. CN, control no HTR; LC, 5% fermented HTRs replaced corn silage; HC, 10% fermented HTRs replaced corn silage.**

### Alternative Diets Containing Fermented HTR Changed the Composition of the Fecal Microbiota

**FIGURE 4:**
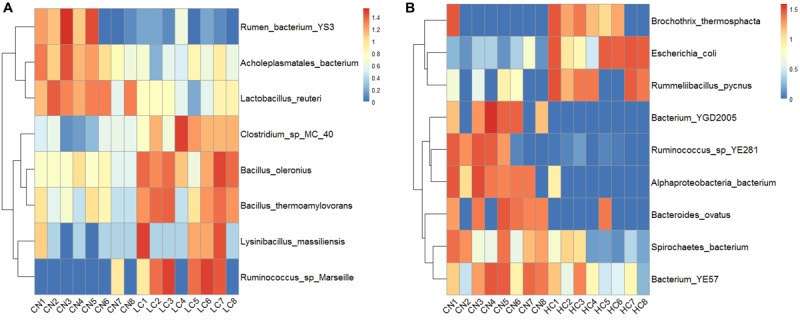
Significant differences between CN and LC groups identified at Species-taxa level **(A)**, and significant differences between CN and HC groups identified at Species-taxa level **(B)**.

The changes in the fecal microbiota at the genus and species levels were determined using a ternary plot method and are shown in Supplementary Figure S3. Compared with the CN and HC groups, the LC group showed a remarkably increased abundance of *Oxyphotobacteria* at the Genus level, while the abundances of *Bifidobacterium*, *Aerosphaera*, *Erysipelothrix*, *Enterobacteriaceae*, *Jeotgalibaca*, and *Acinetobacter* were remarkably enhanced in the HC group. At the species level, the LC group showed significant increases in *Lolium perenne*, *Spirochaetes bacterium*, and *Clostridium* sp. *MC 40*, and the HC group showed significant increases in *Escherichia coli*. *Treponema porcinum* and *Ruminococcus* sp. *YE281* were significantly enhanced in the CN group. The differences in the microbial composition (relative abundance) of the fecal microbiome between the CN and LC groups and the CN and HC groups were compared using the Metastat method with Fisher’s exact test (White et al., 2009). A total of 17 general displayed a significant difference between the CN and LC groups. Among them, *Negativibacillus*, *Eisenbergiella, Anaerovibrio*, *Mollicutes*, *Anaerovorax*, *Mailhella*, *Bacteroidales*, *Flavonifractor*, *Pseudoramibacter*, *Oscillibacter*, *Ruminococcaceae*, and *Butyrivibrio* were decreased, and *Bacillus*, *Akkermansia*, *Parvibacter*, *Turicibacter*, and *Lysinibacillus* were increased in the LC group (Supplementary Table S4A). Compared with the CN group, six genera (*Enterobacteriaceae*, *Brochothrix*, *Akkermansia*, *Rummeliibacillus*, *Campylobacter*, and *Methanocorpusculum*) displayed a significant increase and 11 genera (*Pygmaiobacter*, *Roseburia*, *Rhodospirillales*, *Anaerocolumna*, *Erysipelotrichaceae*, *Eisenbergiella*, *Succinivibrio*, *Butyrivibrio*, *Sanguibacteroides*, *Oxyp*, and *Prevotellaceae*) displayed a significant decrease in the HC group (Supplementary Table S4B). At the species level, a total of eight species displayed a significant difference between the CN and LC groups (Supplementary Table S5A). Among them, *Bacillus oleronius*, *Ruminococcus* sp., *Marseille*, *Bacillus thermoamylovorans*, *Clostridium* sp. *MC 40*, and *Lysinibacillus massiliensis* were upregulated, and *Lactobacillus reuteri*, *Acholeplasmatales bacterium*, and *rumen bacterium YS3* were downregulated (Figure 4A). A total of nine species displayed a significant difference between CN and HC groups (Supplementary Table S5B). Among them, *Alphaproteobacteria bacterium*, *bacterium YGD2005*, *Ruminococcus* sp. *YE281*, *Bacteroides ovatus*, *bacterium YE57*, and *Spirochaetes bacterium* were downregulated, and *Escherichia coli*, *Brochothrix thermosphacta*, and *Rummeliibacillus pycnus* were upregulated (Figure 4B).

### Correlations of Fecal Microbiota With the Energy Utilization Rate and SCFAs

**FIGURE 5:**
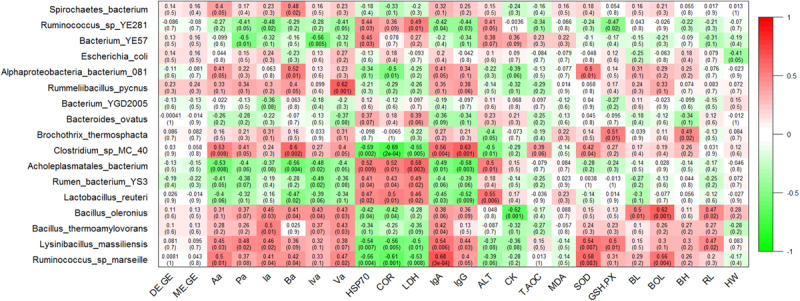
Correlation analyses of species taxa with the energy utilization rate, fecal short chain fatty acid (SCFA) concentrations, serum biochemical indices, and growth performance. Each cell contains the corresponding correlation and *P*-value. The table is color-coded by correlation according to the color legend. DE/GE, the ratio of digestible energy to gross energy; ME/GE, the ratio of metabolizable energy to gross energy; Aa, acetic acid; Pa, propionic acid; Ia, isobutyric acid; Ba, butyric acid; Iva, isovaleric acid; Va, valeric acid. HSP70, Heat shock protein 70; COR, cortisol; LDH, lactate dehydrogenase; IgA, immunoglobulin G; IgG, immunoglobulin G; ALT, alanine aminotransferase; CK, creatine kinase; T-AOC, total antioxidant capacity; MDA, malondialdehyde; SOD, superoxide dismutase; GSH-PX, glutathione peroxidase; BL, body length; BOL, body oblique length; BH, body height; RL, rump length; HW, hip width.

To further identify genera that significantly correlated with the energy utilization rate and the SCFAs concentrations of heifers, we used the Pearson correlation test and found that the bacterial abundance of *Turicibacter* was slightly and positively related to the ratio of DE/GE and ME/GE, but did not reach significance (*P* = 0.09 and *P* = 0.08, respectively) (Supplementary Figure S4). At the genus level, the concentration of Aa, Pa, Ba, and Va displayed strong positive correlations with relative abundance of *Turicibacter* (*P* = 0.009, *P* = 0.04, *P* = 0.01, and *P* = 0.008, respectively). The concentration of Aa, Pa, Ia, Iva, and Va showed significant negative correlations with relative abundance of *Butyrivibrio* (*P* = 0.05, *P* = 0.004, *P* = 0.01, *P* = 0.005, and *P* = 0.004, respectively) (Supplementary Figure S4). At the species level, the relative abundance of *Ruminococcus* sp. *marseille* correlated positively with the concentrations of Aa, Ia, Ba, and Va (*P* = 0.01, *P* = 0.04, *P* = 0.04, and *P* = 0.02, respectively). The relative abundance of *Lysinibacillus massiliensis* correlated positively with the concentrations of Aa, Pa, and Ia (*P* = 0.03, *P* = 0.02, and *P* = 0.02, respectively). In addition, the concentration of Aa and Ba displayed a strong positive correlation with relative abundance of *Clostridium* sp. *MC 40* (*P* = 0.008 and *P* = 0.002, respectively) and a negative correlation with relative abundance of *Acholeplasmatales bacterium* (*P* = 0.008, *P* = 0.004, respectively) (Figure 5).

### Correlations of Fecal Microbiota With Serum Biochemical Indexes

At the genus level, the concentration of HSP70, Cor, and LDH correlated positively with the relative abundance of *Mollicutes* (*P* = 0.01, *P* = 0.01 and *P* = 0.003, respectively) and *Eisenbergiella* (*P* = 0.003, *P* = 0.007 and *P* = 0.002, respectively), while the correlated significantly negatively with the relative abundance of *Lysinibacillus* (*P* = 0.007, *P* = 0.005, and *P* = 0.01, respectively). The concentration of IgA and IgG showed significant positive correlations with relative abundance of *Lysinibacillus* (*P* = 0.006 and *P* = 0.03, respectively) and *Turicibacter* (*P* = 0.03 and *P* = 0.03, respectively), and negative correlations with relative abundance of *Eisenbergiella* (*P* = 0.002 and *P* = 0.002, respectively) and *Negativibacillus* (*P* = 0.001 and *P* = 0.008, respectively). The bacterial abundance of *Butyrivibrio* correlated positively with the concentration of ALT (*P* = 0.002) and CK (*P* = 0.003). Additionally, the bacterial abundance of *Lysinibacillus* correlated positively with the concentration of SOD (*P* = 0.007) and GSH.PX (*P* = 0.01) (Supplementary Figure S4). At the species level, the relative abundance of *Acholeplasmatales bacterium* correlated positively with the concentrations of HSP70, Cor, LDH, and ALT, and correlated significantly negatively with the concentration of IgA and IgG. In contrast, the relative abundance of *Clostridium* sp. *MC 40*, *Lysinibacillus massiliensis*, and *Ruminococcus* sp. *marseille* correlated negatively with the concentrations of HSP70, Cor, and LDH, and correlated positively with the concentrations of IgA and IgG. In addition, the relative abundance of *Lysinibacillus massiliensis* correlated positively with the concentrations of SOD and GSH.PX (Figure 5).

### Correlations of Fecal Microbiota With Growth Performance

At the genus level, the relative abundances of *Bacillus* and *Anaerovibrio* displayed strong positive (Pearson’s *r* = 0.59, *P* = 0.002) and negative correlations (Pearson’s *r* = −0.5, *P* = 0.01) with the BOL value. The RL value correlated positively with the relative abundance of *Lysinibacillus* (Pearson’s *r* = 0.47, *P* = 0.02) and *Bacillus* (Pearson’s *r* = 0.42, *P* = 0.04), and correlated negatively with the relative abundance of *Anaerovibrio* (Pearson’s *r* = −0.49, *P* = 0.01) and *Mailhella* (Pearson’s *r* = −0.48, *P* = 0.02) (Supplementary Figure S4). At the species level, the relative abundances of *Bacillus oleronius* displayed a strong positive correlation with the values of BL, BOL, and RL (*P* = 0.01, *P* = 0.001, and *P* = 0.02, respectively). The relative abundance of *Ruminococcus* sp. *marseille also* showed a strong positive correlation with the BOL value (*P* = 0.004) and the relative abundance of *Lysinibacillus massiliensis* correlated positively with the value of RL (*P* = 0.02) (Figure 5).

## Discussion

Herbal teas have heat-clearing and detoxifying effects and are favored by the population of southern China, leading to gradually increasing demand for herbal teas and the production of massive amounts HTRs. The main herbal tea production enterprises in Guangdong Province can produce about 680 tons of HTRs per day currently, which wastes resources and pollutes the environment. Some studies have shown that tea residues could be used as feed additives (Zhong et al., 2009). However, HTRs are difficult to store because of their high moisture content. Therefore, this study used oat hay to control the moisture content, because it contains high concentrations of non-structural carbohydrates and provides abundant carbohydrates for silage fermentation (Chatterton et al., 2006). In this study, the CP, NDF, and ADF contents (82.3 g/kg of DM, 510.0 g/kg of DM, and 301.2 g/kg of DM, respectively) of the HTRs fermented for 20 days were higher than that of maize straw silage (78.6 g/kg of DM, 462.1 g/kg of DM, and 265.2 g/kg of DM, respectively) (Zeng et al., 2018). Simultaneously, the fermented HTR was well preserved with a low pH and high lactic acid content. Hence, the fermented HTR could be used as good-quality roughage for ruminants. Additionally, HTR fermented with oat hay was used as a partial substitute for corn silage in the feed of Holstein heifers, representing a new use for HTRs as forage for livestock.

An early study suggested that Holstein cows are sensitive to heat stress (HS) conditions when the THI is over 72 (Rhoads et al., 2009). In this study, the average THI was 79.2, which this indicated that the experimental Holstein heifers in South China were substantially affected by HS during summer months. Additionally, the RT and RR are also excellent indicators of an animal’s susceptibility to heat load (Yan et al., 2016). In the presents study, the results showed that both the RT and RR were affected by the fermented HTR, and the optimal dietary supplement was 5% fermented HTRs replaced corn silage, at which the RT and RR of the heifers were the lowest. This finding is consistent with a study conducted by Song et al. (2014), who demonstrated that traditional Chinese medicine reduced the body temperature or maintained the normal temperature of cows in a high temperature environment. The current study demonstrated that the ADFI and BH were greater for the LC and HC groups (*P* < 0.05) compared with those of the CN group. This is in agreement with the results of Wang et al. (2011), who showed that Chinese herbal mixture feed additives had growth-promoting effects in beef cattle. This could be caused by Chinese herbs, which contain abundant protein, carbohydrate, vitamin, fat, and minerals, which can play an important role in the nutritive equilibrium, thus enhancing growth performance (Liu et al., 2011). Currently, there is no information on the effects of fermented HTRs on the energy utilization of ruminants. However, the energy utilization data showed that the fermented HTR had improved the energy utilization efficiency of ruminants. We suspected that this might be caused by a certain effect on microbial fermentation. Studies have shown that microbial fermented feed may provide more rich nutrition, better palatability, increased digestibility, and improved energy efficiency (Niba et al., 2009).

In the present study, the decreased level of HSP70, Cor, and LDH in the serum of the experimental group indicated that consuming fermented HTR might improve the heat stress resistance of heifers. This result corroborates the report of Wang et al. (2011), who observed that a microbially fermented Chinese herbal combination mighty improve the heat stress resistance of Jersey cattle. In addition, the levels of IgA and IgG in the heifers’ serum were significantly increased in the LC and HC groups. Previous studies reported that fermented Chinese herbal medicines improve the immune function in dairy cows under HS conditions (Shan et al., 2018). Interestingly, we also found that the concentrations of ALT and CK in the heifers’ serum decreased significantly. These results indicated that feeding heifers with fermented HTR has important health effects. However, the specific molecular mechanism is unclear. In terms of antioxidant capacity, the levels of SOD and GSH-PX in the heifers’ serum increased significantly. This result illustrates that fermented HTR might exert an antioxidative effect via its antioxidative constituents. Studies have shown that herbal feed with anti-oxidant properties, which can cope with an excess of free radicals produced upon oxidative stress, could be used to alleviate the negative effects of high ambient temperature (Tuzcu et al., 2008; Wang et al., 2008). Based on the above results, we believe that this mighty be related to HTR’s rich and diverse active substances, however, the specific mechanism needs further research.

There is little information on the effects of the fermented HTRs on fecal SCFA concentrations in heifers. In this study, the fermented HTR had a positive effect on the fecal SCFA concentrations of Aa, Pa, Ia, Ba, Iva, and Va. Therefore, we hypothesized that the fermented HTR might improve SCFA metabolism of ruminants under HS conditions via an as-yet-unidentified mechanism. We also investigated the association between fecal microbial composition and the heifers’ characteristics, including energy utilization rate, fecal SCFA concentrations, serum biochemical indexes, and growth performances under HS conditions. We believe that this is the first study to evaluate the difference in the fecal microbiota between heifers with different performances and to explore the correlation of fecal microbiota with the characteristics of Holstein heifers under HS conditions. The Chao1 index indicated that this sequencing depth was sufficient for further analysis. Clustering analysis revealed that the *Firmicute*s and *Bacteroidete*s were the most dominant phyla among the total sequences. which was consistent with studies conducted by Wang et al. (2018). Additionally, in this study, the composition and structure of the fecal microbial community was affected by fermented HTRs. At the genus level, the abundance of *Turicibacter* and *Butyrivibrio* increased and decreased, respectively, in the feces of Holstein heifers fed fermented HTRs. Interestingly, we found that the bacterial abundance of *Turicibacter* was slightly and positively related to the DE/GE and ME/GE ratios. In addition, the relative abundance of *Turicibacter* correlated positively with the fecal concentrations of Aa, Pa, Ba, and Va and the serum concentrations of IgA, IgG, and GSH-PX; and correlated negatively with the serum concentrations of Cor and CK. In contrast, the relative abundance of *Butyrivibrio* correlated negatively with the fecal concentrations of Aa, Pa, Ia, Iva, and Va and the serum concentrations of IgA and IgG; and correlated positively with the concentration of HSP70, LDH, ALT, and CK. A previous study reported that *Turicibacter* deficiency alters the colonic microbiota and results in damage to the normal function of the colonic epithelium (Zenewicz et al., 2013). Moreover, it also associated positively with immunoregulation (Presley et al., 2010). However, *Butyrivibrio* is more involved in epithelial proliferation and diseases (Mao et al., 2015). At the species level, we discovered that *Ruminococcus* sp. *Marseille*, *Clostridium* sp. *MC 40*, and *Lysinibacillus massiliensis* in the LC group were upregulated, and *Acholeplasmatales bacterium* in the LC or HC groups was downregulated compared with those in the CN group. The abundance of *Ruminococcus* sp. *Marseille* correlated positively with the fecal concentration of Aa, Ia, Ba, and Va; the serum concentrations of IgA and IgG; SOD; and the BOL value, and correlated negatively with the serum concentrations of HSP70, Cor, LDH, and ALT. Park et al. (2017) reported that members of the genus *Ruminococcus* protected neurons from oxidative damage by increasing SOD and GSH levels. The abundance of *Lysinibacillus massiliensis* and *Clostridium* sp. *MC 40* correlated positively with the fecal concentrations of Aa, Pa, Ba, and Va; the serum concentrations of IgA, IgG, and SOD; and the BOL value; and correlated negatively with the serum concentrations of HSP70, Cor, LDH, and ALT. The bacterial abundance of *Acholeplasmatales bacterium* correlated negatively with the fecal concentrations of Aa, Ba, Iva and Va; and the serum concentrations of IgA and IgG; and correlated positively with the serum concentrations of HSP70, Cor, LDH, and ALT. Recent studies have revealed that members of the species *Lysinibacillus* have potential antimicrobial properties against foodborne bacterial and fungal pathogens (Ahmad et al., 2014; Seelam et al., 2018). Zeng et al. (2011) reported that a decrease in *Clostridium* resulted in enterotoxemic diseases and other colonic epithelium diseases. However, *Acholeplasmatales bacterium* was established as pathogenic by Nicolet (1996). In addition, a study reported that the relative abundance of *Acholeplasmatales* also correlated with intestinal inflammation (Dicksved et al., 2015). Therefore, a hypothesis was proposed that fermented HTRs mighty improve growth traits and energy utilization efficiency, increased antioxidant capacity, enhance immunity, and relieve HS by altering the microbial communities. However, more in-depth and detailed research is needed to confirm this hypothesis.

## Conclusion

The results of the present study indicated that fermented HTRs promoted physiological traits, increased growth performance, improved energy utilization efficiency, enhanced immunity, strengthened antioxidant function, and mitigated heat stress in Holstein heifers. Additionally, we also detailed the fecal microbial community composition of Holstein heifers in response to HTRs. The bacteria are closely related to characteristics including the energy utilization rate, growth performance, serum biochemical indexes, and fecal SCFA levels of the heifers. Based on our findings, the 5% fermented HTRs replaced corn silage might be advantageous for the heifers’ characteristics. This might be related to the inclusion of a variety of biologically active substances; however, the specific mechanism requires further research.

## Data Availability Statement

All datasets generated for this study are included in the article/Supplementary Material.

## Ethics Statement

The animal study was reviewed and approved by South China Agricultural University.

## Author Contributions

YX and JS led the lab assays, analyses of data, and writing of the manuscript. JS and YZ contributed reagents, materials, and analysis tools. All authors gave final approval for publication and were involved in project conception and design.

## Conflict of Interest

The authors declare that the research was conducted in the absence of any commercial or financial relationships that could be construed as a potential conflict of interest.

## Footnotes

1 https://www.ncbi.nlm.nih.gov/sra/

2 http://horvath.genetics.ucla.edu/html/CoexpressionNetwork/Rpackages/WGCNA/

## Supplementary Material

FIGURE S1 Changes in the temperature humidity index (THI) at different times of day in the cowshed during the experimental period.

FIGURE S2 Rarefaction curves. Number of sequences **(A)** and number of sample **(B)** rarefaction curves for the sampled fecal microbiotas. Number of detected OTUs on the y-axis; number of sequences **(A)** and of samples **(B)** on the x-axis.

FIGURE S3 Ternary plot showing an abundance comparison of the fecal microbiota among the three groups at genus-taxa and species-taxa levels.

FIGURE S4 Correlation analyses of genus-taxa with the energy utilization rate, fecal SCFA concentrations, serum biochemical indexes, and growth performance. Each cell contains the corresponding correlation and *P*-value. The table is color-coded by correlation according to the color legend. DE/GE, the ratio of digestible energy to gross energy; ME/GE, the ratio of metabolizable energy to gross energy; Aa, acetic acid; Pa, propionic acid; Ia, isobutyric acid; Ba, butyric acid; Iva, isovaleric acid; Va, valeric acid. HSP70, heat shock protein 70, COR: cortisol; LDH, lactate dehydrogenase; IgA, immunoglobulin G; IgG, immunoglobulin G; ALT, alanine aminotransferase; CK, creatine kinase; T-AOC, total antioxidant capacity; MDA, malondialdehyde; SOD, superoxide dismutase; GSH-PX, glutathione peroxidase; BL, body length; BOL, body oblique length; BH, body height; RL, rump length; HW, hip width.

TABLE S1 Nutritional composition of HTRs; pH and nutrient composition of HTRs before and after fermentation; ingredients of the concentrate formula; ingredients and nutrient compositions of the total mixed rations in the three treatments.

TABLE S2A Quality control and preprocessing of metagenomic datasets.

TABLE S2B Sequence composition of each sample at each level in the Greengenes database.

TABLE S2C Aligned counts at each level between different samples.

TABLE S3A The aligned counts that were annotated at the phylum level.

TABLE S3B The aligned percentages that were annotated at the phylum level.

TABLE S3C The aligned counts that were annotated at the class level.

TABLE S3D The aligned percentages that were annotated at the class level.

TABLE S3E The aligned counts that were annotated at the order level.

TABLE S3F The aligned percentages that were annotated at the order level.

TABLE S3G The aligned counts that were annotated at the family level.

TABLE S3H The aligned percentages that were annotated at the family level.

TABLE S3I The aligned counts that were annotated at the genus level.

TABLE S3J The aligned percentages that were annotated at the genus level.

TABLE S3K The aligned counts that were annotated at the species level.

TABLE S3L The aligned percentages that were annotated at the species level.

TABLE S4A Significant differences between the CN and LC groups identified at the genus-taxa level.

TABLE S4B Significant differences between the CN and HC groups identified at the genus-taxa level.

TABLE S5A Significant differences between the CN and LC groups identified at the species-taxa level.

TABLE S5B Significant differences between the CN and HC groups identified at the species-taxa level.

## References

[B1] Abd A. E. A. Choi J. H. Rahman M. M. Kim S. W. Tosun A. Shim J. H. (2014). Residues and contaminants in tea and tea infusions: a review. Food additives & contaminants. *Part A Chem. Analy. Control Expos. Risk Assess.* 31 1794–1804. 10.1080/19440049.2014.958575 25164107

[B2] Ahmad V. Iqbal A. M. Z. Haseeb M. Khan M. S. (2014). Antimicrobial potential of bacteriocin producing *Lysinibacillus* jx416856 against foodborne bacterial and fungal pathogens, isolated from fruits and vegetable waste. *Anaerobe* 27 87–95. 10.1016/j.anaerobe.2014.04.001 24735603

[B3] Ahsan M. A. Katla S. K. Islam M. T. Hernandez-Viezcas J. A. Martinez L. M. Díaz-Moreno C. A. (2018). Adsorptive removal of methylene blue, tetracycline and Cr (VI) from water using sulfonated tea waste. *Environ. Technol. Innovat.* 11 23–40. 10.1016/j.eti.2018.04.003

[B4] Barker S. B. Summerson W. H. (1941). The colorimetric determination of lactic acid in biological material. *J. Biol. Chem.* 138 535–554.

[B5] Chatterton N. J. Watts K. A. Jensen K. B. Harrison P. A. Horton W. H. (2006). Nonstructural carbohydrates in oat forage. *J. Nutrit.* 136:2111S 10.1093/jn/136.7.2111S 16772513

[B6] Chemists A. A. Horwitz W. (1990). *Official Methods Of Analysis*, 15th Edn, Arlington, VA: AOAC.

[B7] Dicksved J. Jansson J. K. Lindberg J. E. (2015). Fecal microbiome of growing pigs fed a cereal based diet including chicory (*Cichorium intybus* L.) or ribwort (*Plantago lanceolata* L.) forage. *J. Anim. Sci. Biotechnol.* 6:53 10.1186/s40104-015-0054-8 PMC4683726 26688727

[B8] Edgar R. C. (2004). MUSCLE: multiple sequence alignment with high accuracy and high throughput. *Nucleic Acids Res.* 32 1792–1797. 10.1093/nar/gkh340 15034147 PMC390337

[B9] Edgar R. C. (2013). UPARSE: highly accurate OTU sequences from microbial amplicon reads. *Nat. Methods* 10:996 10.1038/nmeth.2604 23955772

[B10] Edgar R. C. Haas B. J. Clemente J. C. Quince C. Knight R. (2011). UCHIME improves sensitivity and speed of chimera detection. *Bioinformatics* 27 2194–2200. 10.1093/bioinformatics/btr381 21700674 PMC3150044

[B11] Fang J. Cao Y. Matsuzaki M. Suzuki H. (2016). Effects of apple pomace proportion levels on the fermentation quality of total mixed ration silage and its digestibility, preference and ruminal fermentation in beef cows. *Anim. Sci. J.* 87 217–223. 10.1111/asj.12410 26278555

[B12] Gurung M. Adhikari B. B. Alam S. Kawakita H. Ohto K. Inoue K. (2013). Adsorptive removal of Cs (I) from aqueous solution using polyphenols enriched biomass-based adsorbents. *Chem. Eng. J.* 231 113–120. 10.1016/j.cej.2013.06.028

[B13] Haas B. J. Gevers D. Earl A. M. Feldgarden M. Ward D. V. Giannoukos G. (2011). Chimeric 16S rRNA sequence formation and detection in Sanger and 454-pyrosequenced PCR amplicons. *Genome Res.* 21 494–504. 10.1101/gr.112730.110 21212162 PMC3044863

[B14] Horwitz W. (2010). “Official methods of analysis of AOAC international,” in *Agricultural Chemicals Contaminants Drugs*, ed. Horwitz W. (Gaithersburg: AOAC International).

[B15] Iqbal Khan M. A. Ueno K. Horimoto S. Komai F. Tanaka K. Ono Y. (2007). Evaluation of the physio-chemical and microbial properties of green tea waste-rice bran compost and the effect of the compost on spinach production. *Plant Product. Sci.* 10 391–399. 10.1626/pps.10.391

[B16] Kim D. G. Lee M. R. Yoo J. M. Park K. I. Ma J. Y. (2017). Fermented herbal formula KIOM-MA-128 protects against acute colitis induced by dextran sodium sulfate in mice. *BMC Complement. Alternat. Med.* 17:354 10.1186/s12906-017-1855-4 PMC5499052 28679372

[B17] Kovács L. Kézér F. L. Ruff F. Jurkovich V. Szenci O. (2018). Heart rate, cardiac vagal tone, respiratory rate, and rectal temperature in dairy calves exposed to heat stress in a continental region. *Intern. J. Biometeorol.* 62 1791–1797. 10.1007/s00484-018-1581-8 30032363

[B18] Kumar S. Bass B. E. Bandrick M. Loving C. L. Brockmeier S. L. Looft T. (2017). Fermentation products as feed additives mitigate some ill-effects of heat stress in pigs. *J. Anim. Sci.* 95 279–290. 10.2527/jas2016.0662 28177370

[B19] Li D. L. Zheng X. L. Duan L. Deng S. W. Ye W. Wang A. H. (2017). Ethnobotanical survey of herbal tea plants from the traditional markets in Chaoshan. China. *J. Ethnopharmacol.* 205 195–206. 10.1016/j.jep.2017.02.040 28249822

[B20] Liu H. W. Tong J. M. Zhou D. W. (2011). Utilization of Chinese herbal feed additives in animal production. *Agric. Sci. China* 10 1262–1272. 10.1016/S1671-2927(11)60118-1

[B21] Mao S. Zhang M. Liu J. Zhu W. (2015). Characterising the bacterial microbiota across the gastrointestinal tracts of dairy cattle: membership and potential function. *Sci. Rep.* 5:16116 10.1038/srep16116 PMC4630781 26527325

[B22] Martin M. (2011). Cutadapt removes adapter sequences from high-throughput sequencing reads. *EMBnet J.* 17 10–12. 10.14806/ej.17.1.200

[B23] Naderi N. Ghorbani G. R. Sadeghi-Sefidmazgi A. Nasrollahi S. M. Beauchemin K. A. (2016). Shredded beet pulp substituted for corn silage in diets fed to dairy cows under ambient heat stress: Feed intake, total-tract digestibility, plasma metabolites, and milk production. *J. Dairy Sci.* 99 8847–8857. 10.3168/jds.2016-11029 27592434

[B24] Niba A. T. Beal J. D. Kudi A. C. Brooks P. H. (2009). Potential of bacterial fermentation as a biosafe method of improving feeds for pigs and poultry. *Afr. J. Biotechnol.* 8 1758–1767. 10.4314/ajb.v8i9.60378

[B25] Nicolet J. (1996). Animal mycoplasmoses: a general introduction. *Revue Sci. Techn. Off. Intern. Epizoot.* 15 1233–1240. 10.20506/rst.15.4.982 9190015

[B26] Ozkaya S. Bozkurt Y. (2008). The relationship of parameters of body measures and body weight by using digital image analysis in pre-slaughter cattle. *Archiv. Anim. Breed.* 51 120–128. 10.5194/aab-51-120-2008

[B27] Pardau M. D. Pereira A. S. Apostolides Z. Serem J. C. Bester M. J. (2017). Antioxidant and anti-inflammatory properties of Ilex guayusa tea preparations: a comparison to *Camellia sinensis* teas. *Food Funct.* 8 4601–4610. 10.1039/C7FO01067B 29134218

[B28] Park J. Lee J. Yeom Z. Heo D. Lim Y. H. (2017). Neuroprotective effect of *Ruminococcus albus* on oxidatively stressed SH-SY5Y cells and animals. *Sci. Rep.* 7:14520 10.1038/s41598-017-15163-5 PMC5674049 29109537

[B29] Presley L. L. Wei B. Braun J. Borneman J. (2010). Bacteria associated with immunoregulatory cells in mice. *Appl. Environ. Microbiol.* 76 936–941. 10.1128/AEM.01561-09 20008175 PMC2813032

[B30] Quast C. Pruesse E. Yilmaz P. Gerken J. Schweer T. Yarza P. (2012). The SILVA ribosomal RNA gene database project: improved data processing and web-based tools. *Nucleic Acids Res.* 41 D590–D596. 10.1093/nar/gks1219 23193283 PMC3531112

[B31] Rhoads M. L. Rhoads R. P. VanBaale M. J. Collier R. J. Sanders S. R. Weber W. J. (2009). Effects of heat stress and plane of nutrition on lactating holstein cows: i. production metabolism and aspects of circulating somatotropin. *J. Dairy Sci.* 92 1986–1997. 10.3168/jds.2008-1641 19389956

[B32] Seelam N. S. Katike U. Kotha P. Akula H. Obulam V. S. R. (2018). Hypolipidemic effects of Lysinibacillus sphaericus fermented tomato and carrot juices in high-fat diet-fed albino Wistar rats. *J. Appl. Biol. Biotechnol.* 6 64–70. 10.7324/JABB.2018.60611

[B33] Shan C. H. Guo J. Sun X. Li N. Yang X. Gao Y. (2018). Effects of fermented Chinese herbal medicines on milk performance and immune function in late-lactation cows under heat stress conditions. *J. Anim. Sci.* 96 4444–4457. 10.1093/jas/sky270 30032262 PMC6162570

[B34] Shen B. Tian L. Li F. Zhang X. Xu H. Singh S. (2017). Elemental mercury removal by the modified bio-char from waste tea. *Fuel* 187 189–196. 10.1016/j.fuel.2016.09.059

[B35] Song X. Luo J. Fu D. Zhao X. Bunlue K. Xu Z. (2014). Traditional Chinese medicine prescriptions enhance growth performance of heat stressed beef cattle by relieving heat stress responses and increasing apparent nutrient digestibility. *Asian Austr. J. Anim. Sci.* 27:1513 10.5713/ajas.2014.14058 PMC4150185 25178304

[B36] Srikandakumar A. Johnson E. H. (2004). Effect of heat stress on milk production, rectal temperature, respiratory rate and blood chemistry in Holstein, Jersey and Australian Milking Zebu cows. *Trop. Anim. Health Product.* 36 685–692. 10.1023/B:TROP.0000042868.76914.a9 15563029

[B37] Su J. Zhu Q. Zhao Y. Han L. Yin Y. Blachier F. (2018). Dietary supplementation with chinese herbal residues or their fermented products modifies the colonic microbiota, bacterial metabolites, and expression of genes related to colon barrier function in weaned piglets. *Front. Microbiol.* 9:3181 10.3389/fmicb.2018.03181 PMC6309725 30627122

[B38] Sun J. Zeng B. Chen Z. Yan S. Huang W. Sun B. (2017). Characterization of faecal microbial communities of dairy cows fed diets containing ensiled *Moringa oleifera* fodder. *Sci. Rep.* 7:41403 10.1038/srep41403 PMC5278366 28134261

[B39] Tuzcu M. Sahin N. Karatepe M. Cikim G. Kilinc U. Sahin K. (2008). Epigallocatechin-3-gallate supplementation can improve antioxidant status in stressed quail. *Br. Poult. Sci.* 49 643–648. 10.1080/00071660802298336 18836912

[B40] Van Keulen J. Young B. A. (1977). Evaluation of acid-insoluble ash as a natural marker in ruminant digestibility studies. *J. Anim. Sci.* 44, 282–287. 10.2527/jas1977.442282x

[B41] Van Soest P. V. Robertson J. B. Lewis B. A. (1991). Methods for dietary fiber, neutral detergent fiber, and nonstarch polysaccharides in relation to animal nutrition. *J. Dairy Sci.* 74 3583–3597. 10.3168/jds.S0022-0302(91)78551-2 1660498

[B42] Wang H. Ji Y. Yin C. Deng M. Tang T. Deng B. (2018). Differential analysis of gut microbiota correlated with oxidative stress in sows with high or low litter performance during lactation. *Front. Microbiol.* 9:1665 10.3389/fmicb.2018.01665 PMC6103269 30154758

[B43] Wang H. F. Yang W. R. Wang Y. X. Yang Z. B. Cui Y. H. (2011). The study on the effects of Chinese herbal mixtures on growth, activity of post-ruminal digestive enzymes and serum antioxidant status of beef cattle. *Agric. Sci. China* 10 448–455. 10.1016/S1671-2927(11)60024-2

[B44] Wang L. Piao X. L. Kim S. W. Piao X. S. Shen Y. B. Lee H. S. (2008). Effects of Forsythia suspensa extract on growth performance, nutrient digestibility, and antioxidant activities in broiler chickens under high ambient temperature. *Poult. Sci.* 87 1287–1294. 10.3382/ps.2008-00023 18577607

[B45] White J. R. Nagarajan N. Pop M. (2009). Statistical methods for detecting differentially abundant features in clinical metagenomic samples. *PLoS Comput. Biol.* 5:e1000352 10.1371/journal.pcbi.1000352 PMC2661018 19360128

[B46] Yan F. Xue B. Song L. Dalecuo J. Xiao J. Ding S. (2016). Effect of dietary net energy concentration on dry matter intake and energy partition in cows in mid-lactation under heat stress. *Anim. Sci. J.* 87 1352–1362. 10.1111/asj.12561 26875539

[B47] Yang X. Cui X. (2013). Adsorption characteristics of Pb (II) on alkali treated tea residue. *Water Resour. Indust.* 3 1–10. 10.1016/j.wri.2013.05.003

[B48] Zenewicz L. A. Yin X. Wang G. Elinav E. Hao L. Zhao L. (2013). IL-22 deficiency alters colonic microbiota to be transmissible and colitogenic. *J. Immunol.* 190 5306–5312. 10.4049/jimmunol.1300016 23585682 PMC3646987

[B49] Zeng B. Sun J. J. Chen T. Sun B. L. He Q. Chen X. Y. (2018). Effects of Moringa oleifera silage on milk yield, nutrient digestibility and serum biochemical indexes of lactating dairy cows. *J. Anim. Physiol. Anim. Nutr.* 102 75–81. 10.1111/jpn.12660 28299866

[B50] Zeng J. Deng G. Wang J. Zhou J. Liu X. Xie Q. (2011). Potential protective immunogenicity of recombinant Clostridium perfringens α–β2–β1 fusion toxin in mice, sows and cows. *Vaccine* 29 5459–5466. 10.1016/j.vaccine.2011.05.059 21641956

[B51] Zhong R. Z. Tan C. Y. Han X. F. Tang S. X. Tan Z. L. Zeng B. (2009). Effect of dietary tea catechins supplementation in goats on the quality of meat kept under refrigeration. *Small Rumin. Res.* 87 122–125. 10.1016/j.smallrumres.2009.10.012

[B52] Zou C. X. Lively F. O. Wylie A. R. G. Yan T. (2016). Estimation of the maintenance energy requirements, methane emissions and nitrogen utilization efficiency of two suckler cow genotypes. *Animal* 10 616–622. 10.1017/S1751731115002268 26593693

