# Supplementary figures and images for: Effects of Fermented Herbal Tea Residues on the Intestinal Microbiota Characteristics of Holstein Heifers Under Heat Stress
Source: Front Microbiol. 2020 May 26;11:1014. doi: 10.3389/fmicb.2020.01014 (PMC7264259; doi:10.3389/fmicb.2020.01014)

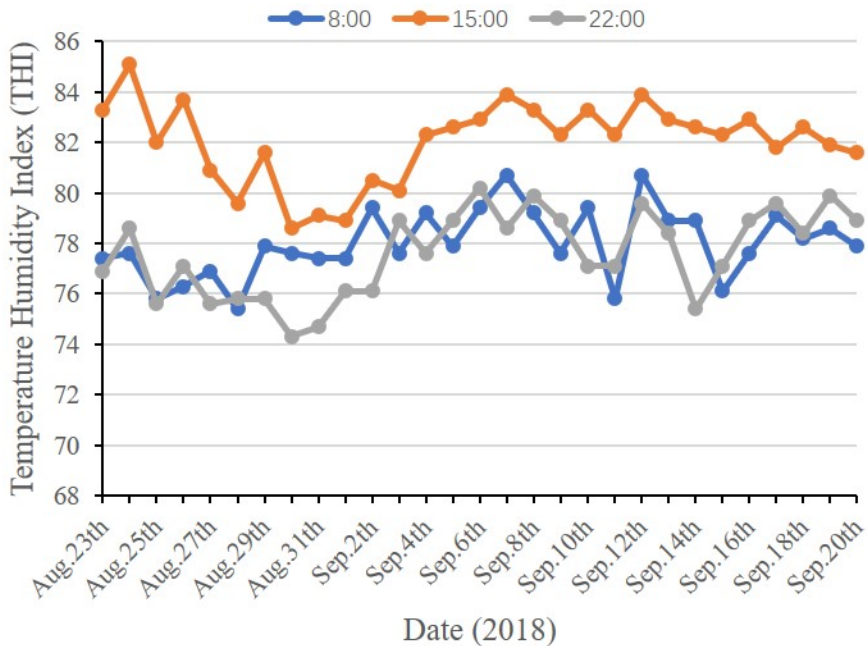

Supplement: FIGURE S1 — Changes in the temperature humidity index (THI) at different times of day in the cowshed during the experimental period. [file Data_Sheet_1.zip › Figure S1.pdf]

**A**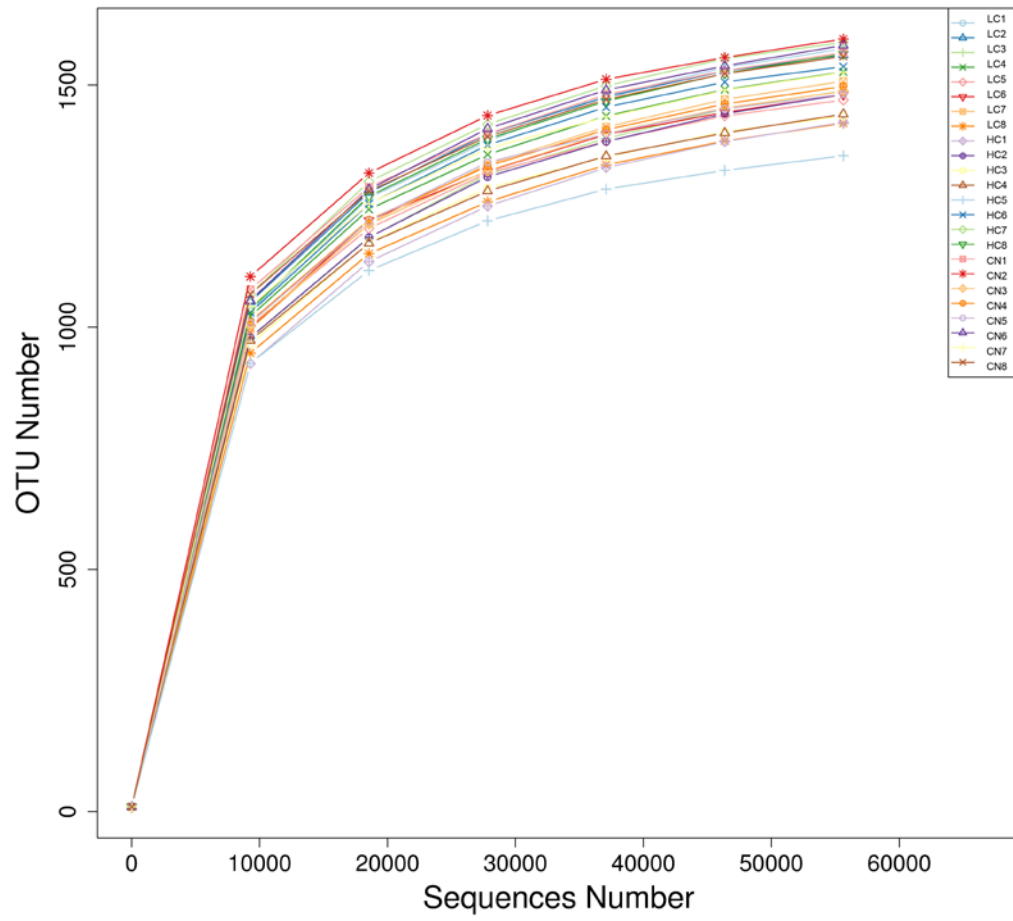**B**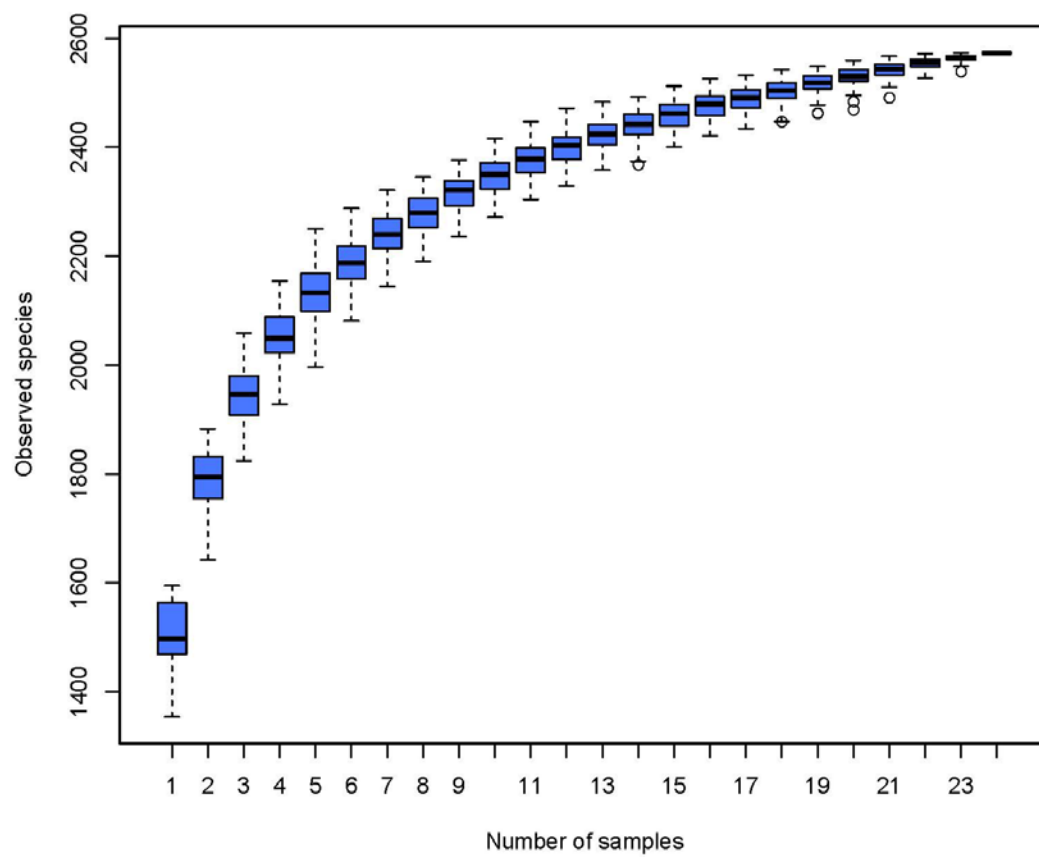

Supplement: FIGURE S1 — Changes in the temperature humidity index (THI) at different times of day in the cowshed during the experimental period. [file Data_Sheet_1.zip › Figure S2.pdf]

# Ternaryplot

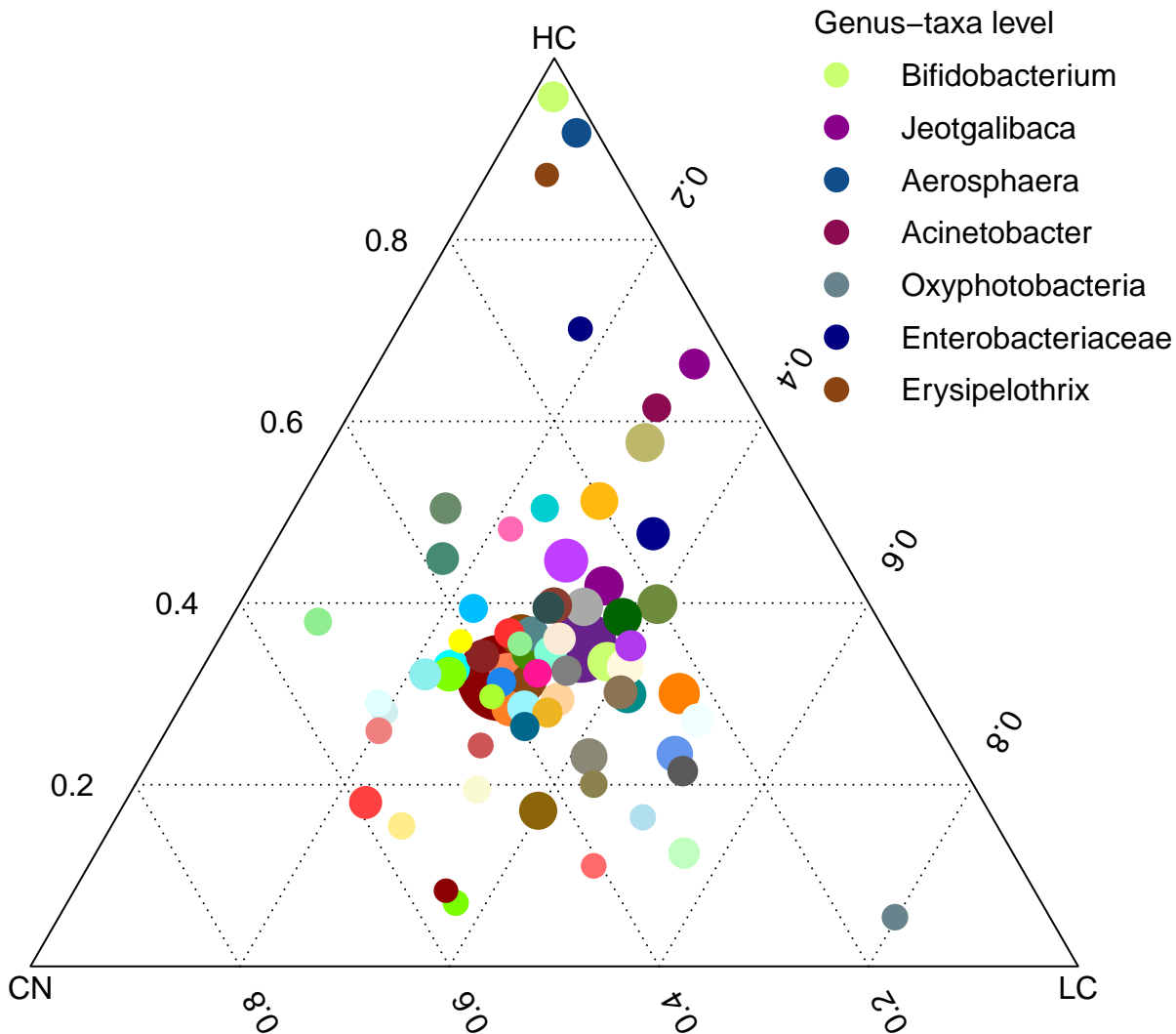

# Ternaryplot

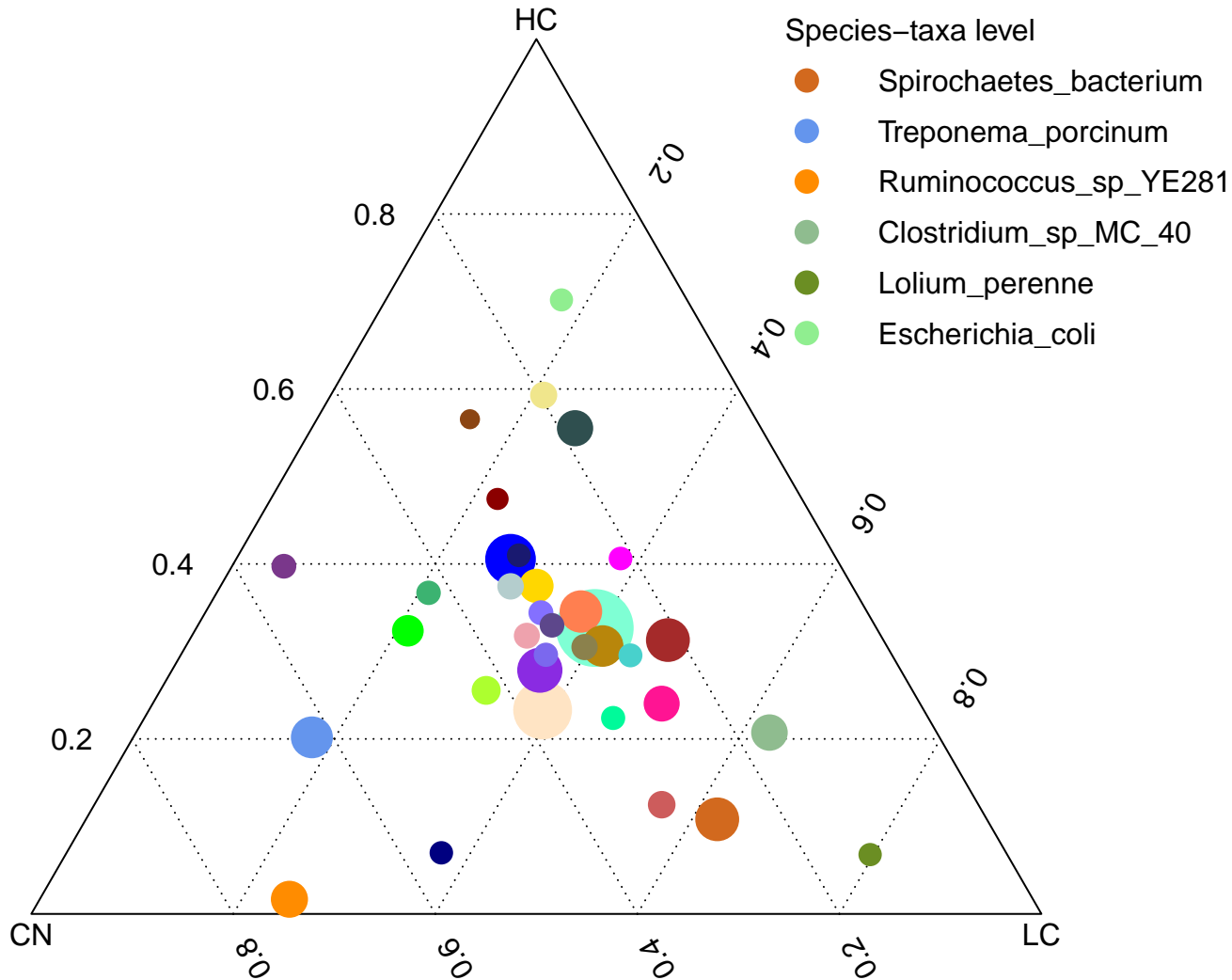

Supplement: FIGURE S1 — Changes in the temperature humidity index (THI) at different times of day in the cowshed during the experimental period. [file Data_Sheet_1.zip › Figure S3.pdf]

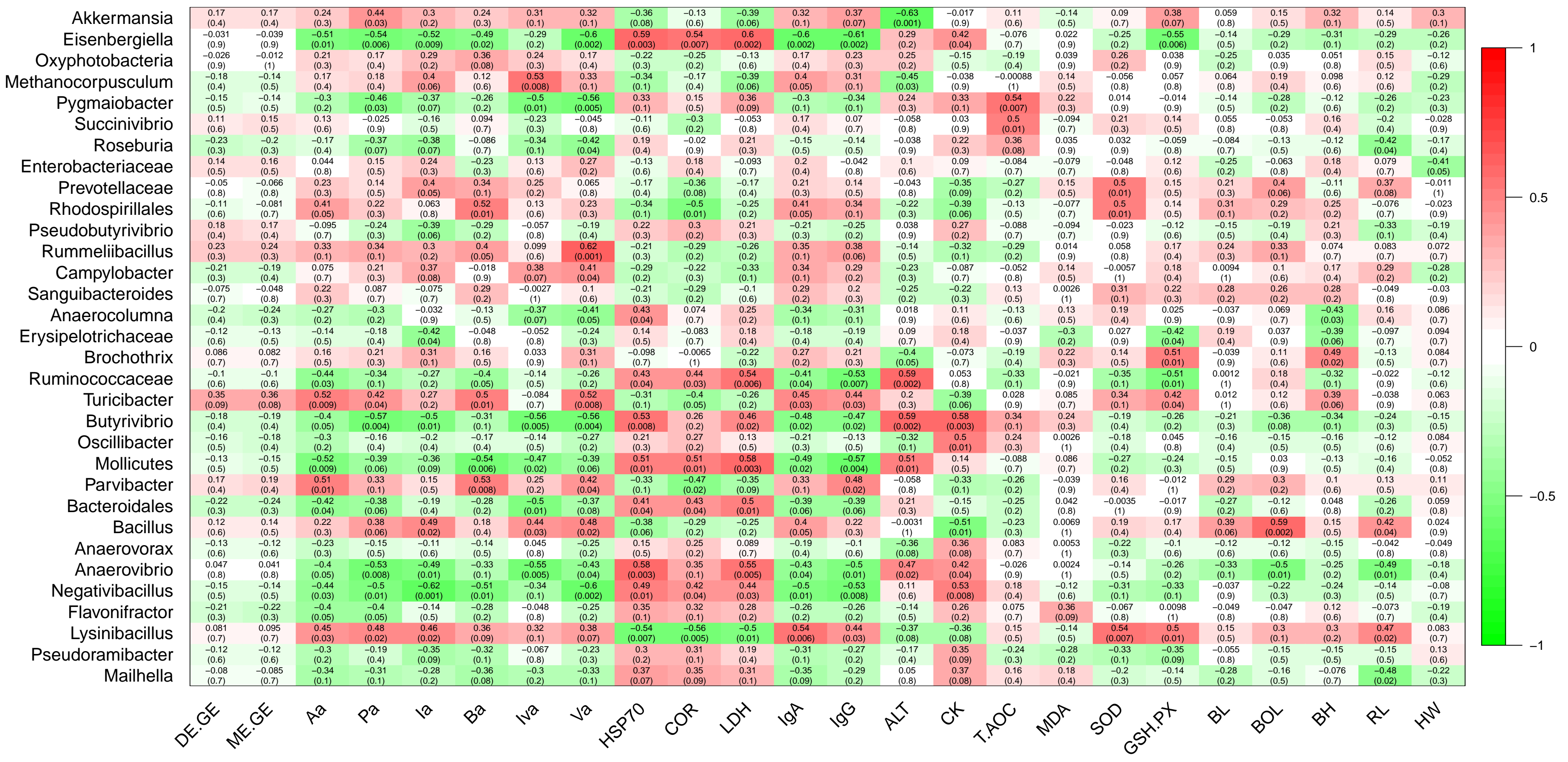

Supplement: FIGURE S1 — Changes in the temperature humidity index (THI) at different times of day in the cowshed during the experimental period. [file Data_Sheet_1.zip › Figure S4.pdf]
