# Supplementary material for: Effects of Fermented Herbal Tea Residues on the Intestinal Microbiota Characteristics of Holstein Heifers Under Heat Stress
Source: Front Microbiol. 2020 May 26;11:1014. doi: 10.3389/fmicb.2020.01014 (PMC7264259; doi:10.3389/fmicb.2020.01014)
Supplement: TABLE S1 — Nutritional composition of HTRs; pH and nutrient composition of HTRs before and after fermentation; ingredients of the concentrate formula; ingredients and nutrient compositions of the total mixed rations in the three treatments. [file Data_Sheet_2.zip › Table S1.docx]

**Nutritional composition of herbal tea residue (HTR) (N = 6)**

| **Ingredient** | **Content (g/kg of dry matter)** |
| --- | --- |
| Dry matter (g/kg of wet) | 205.88 ± 6.75 |
| Crude protein | 97.75 ± 4.32 |
| Crude fat | 35.17 ± 3.03 |
| Crude fiber | 288.28 ± 6.29 |
| Ash | 66.85 ± 4.18 |
| Neutral detergent fiber | 612.18 ± 11.86 |
| Acid detergent fiber | 452.88 ± 12.22 |

**Nutrient composition and pH herbal tea residue (HTR) before and after silage (N = 6)**

| **Ingredient** | **HTR (g/kg of Dry matter)** | |
| --- | --- | --- |
|  | **Zero silage** | **20-day silage** |
| pH (WE*) | 5.60 ± 0.04 | 3.72±0.05 |
| Acetic acid | - | 21.09 ± 0.71 |
| Dry matter (g/kg of wet) | 441.0 ± 2.1 | 436.2 ± 9.49 |
| Crude protein | 79.2 ± 0.6 | 82.3 ± 1.77 |
| Ash | 50.0 ± 0.2 | 55.0 ± 1.01 |
| Neutral detergent fiber | 567.8 ± 2.0 | 510.0 ± 12.98 |
| Acid detergent fiber | 332.0 ± 2.7 | 301.2 ± 8.06 |

Note: * water extract of silage (20 g of HRS and 180 mL of deionized water).

**Ingredients of the concentrate formula**

| **Ingredient** | **Content (g/kg of air dry basis)** |
| --- | --- |
| Corn grain | 380 |
| Distillers Dried Grains with Solubles | 300 |
| Bean pulp | 120 |
| Rapeseed meal | 170 |
| Calcium hydrogen phosphate | 7.4 |
| Calcium carbonate | 7.5 |
| Mineral and vitamin premix* | 6.3 |
| Salt | 8.8 |
| Total | 1000 |

Note: *Contained vitamin A (1,200,000 IU/kg), vitamin D (90,000 IU/kg), vitamin E (8,000 mg/kg), Fe (3,600 mg/kg), Cu (1,600 mg/kg), Mn (3,550 mg/kg), Zn (4,025 mg/kg), Co (30 mg/kg), I (800 mg/kg), Se (70 mg/kg).

**Ingredient and nutrient compositions of total mixed rations in the three treatments (N = 6)**

| **Item** | **Groups (g/kg Total mixed rations)** | | | |
| --- | --- | --- | --- | --- |
|  | **CN** | **LC** | | **HC** |
| Ingredients | | | | |
| Concentrate formula | 290 | 290 | 290 | |
| *Leymus chinensi*s | 150 | 150 | 150 | |
| Corn silage | 560 | 510 | 460 | |
| HTR | 0 | 50 | 100 | |
| Total | 1000 | 1000 | 1000 | |
| Nutrient composition | | | | |
| Dry matter (g/kg of feed) | 554.8 | 560.2 | 563.8 | |
| Crude protein | 130.1 | 128.2 | 127.9 | |
| Neutral detergent fiber | 467.2 | 473.1 | 479.8 | |
| Acid detergent fiber | 220.9 | 235.8 | 241.9 | |
| Ca | 5.8 | 5.6 | 5.6 | |
| P | 4 | 3.9 | 3.9 | |

Note: CN, control no HTR; LC, 5% fermented HTRs replaced corn silage; HC, 10% fermented HTRs replaced corn silage; HTR, herbal tea residue.

**Effects of feeding fermented herbal tea residues on nutrient apparent digestibility of Holstein heifers (%)**

| **Item** | **Groups** | | |
| --- | --- | --- | --- |
|  | **CN** | **LC** | **HC** |
| Dry matter | 61.3 ± 0.9 | 62.5 ± 0.6 | 60.4 ± 1.0 |
| Crude protein | 60.2 ± 1.1 | 61.2 ± 0.8 | 59.6 ± 1.3 |
| Neutral detergent fibre | 54.8 ± 0.6 | 55.7 ± 0.8 | 56.6 ± 0.7 |
| Acid detergent fibre | 46.3 ± 0.6 | 472 ± 0.5 | 487 ± 1.2 |

Note: CN, control no HTR; LC, 5% fermented HTRs replaced corn silage; HC, 10% fermented HTRs replaced corn silage.
